# 5-Hydroxymethylcytosine: a key epigenetic mark in cancer and chemotherapy response

**DOI:** 10.1186/s13072-025-00636-z

**Published:** 2025-11-17

**Authors:** Suhas S. Kharat, Shyam K. Sharan

**Affiliations:** 1 Department of Biochemistry, Vanderbilt University, Nashville, TN 37240 USA; 2 Mouse Cancer Genetics Program, Center for Cancer Research, National Cancer Institute, Frederick, MD 21702 USA

**Keywords:** Epigenetic modification, 5-hydroxymethylcytosine (5hmC), 5-methylcytosine (5mC), Ten-eleven translocation (TET), Biomarker, α-Ketoglutarate (α-KG), Carcinogenesis, Cell-free DNA (cfDNA)

## Abstract

5-hydroxymethylcytosine (5hmC), an epigenetic modification derived from the oxidation of 5-methylcytosine (5mC) by the ten-eleven translocation (TET) family of dioxygenases, plays a pivotal role in the regulation of gene expression, cellular differentiation, and developmental plasticity. Once considered an intermediate in DNA demethylation, 5hmC is now recognized as a stable and functionally significant epigenetic mark with distinct genomic distributions and significant regulatory implications. This review provides a comprehensive analysis of the biological functions of 5hmC in normal cellular processes, including its role in maintaining tissue-specific gene expression, lineage commitment, and genomic integrity. We also describe its role in cancer, the mechanistic underpinnings of its loss or redistribution in tumor cells, and how these changes contribute to oncogenic signaling pathways, epithelial-mesenchymal transition, and tumor heterogeneity. Furthermore, we explore the utility of 5hmC as a biomarker in cancer diagnostics and prognostics, supported by recent advances in sequencing technologies and cell-free DNA profiling. We also examine the intersection of 5hmC and chemotherapy, highlighting how aberrant 5hmC levels can influence drug resistance and sensitivity, and assess the therapeutic potential of targeting TET enzymes and associated pathways. By integrating insights from basic epigenetics, cancer biology, and therapeutic research, this review underscores the multifaceted role of 5hmC in human malignancies and outlines the translational opportunities for exploiting 5hmC-related mechanisms in precision oncology.

## Introduction

The epigenome plays a central role in determining cellular identity, maintaining genomic stability, and regulating gene expression without altering the underlying DNA sequence. Among the various epigenetic modifications, DNA methylation of cytosine has long been recognized as a key modulator of chromatin structure and transcriptional activity. More recently, the discovery of 5hmC, a stable and functionally significant oxidized form of 5mC, has opened new avenues in epigenetics research. 5mC is converted to 5hmC by the catalytic activity of the ten-eleven translocation (TET) family of dioxygenases (TET1, TET2, and TET3) [30, 77]. Initially identified as an intermediate in active DNA demethylation, 5hmC is now recognized for its independent regulatory functions in diverse biological contexts, including embryonic development, neurogenesis, hematopoiesis, and stem cell pluripotency [83]. The presence and distribution of 5hmC are dynamically regulated across tissues and developmental stages, and emerging evidence underscores its importance in modulating enhancer activity, promoter function, and the stability of transcriptional programs. Perturbations in 5hmC patterns are increasingly implicated in pathological conditions, most notably cancer. Tumors frequently exhibit global loss of 5hmC, which correlates with disease aggressiveness and poor prognosis. Additionally, mutations in TET genes and their metabolic cofactors (e.g., IDH1/2) further contribute to 5hmC dysregulation [29]. This review explores the multifaceted roles of 5hmC in normal and malignant cells, highlighting its mechanistic contributions to oncogenesis, tumor progression, and therapy resistance. We also examine the translational implications of 5hmC as a diagnostic biomarker and a potential therapeutic target, especially in the context of chemotherapeutic response modulation.

## Biological background of 5hmC

5hmC was first discovered in 1952 in bacteriophage DNA by Wyatt and Cohen, marking the earliest recognition of this modified base in biological systems [84]. However, its presence in mammalian cells and biological significance remained elusive until 2009, when it was definitively identified using mass spectrometry and immunodetection techniques sparking intense interest in its biological functions [77]. In mammalian cells, 5hmC is generated from 5mC through a single TET-mediated oxidation step, which can be further oxidized to 5-formylcytosine (5fC) and 5-carboxylcytosine (5caC) primarily at CpG islands using α-ketoglutarate (α-KG), Fe(II), and molecular oxygen as essential cofactors [28, 31, 83]. These oxidized derivatives are excised by thymine DNA glycosylase (TDG) and repaired through the base excision repair pathway, thereby completing the active DNA demethylation process [11, 28, 55]. 5mC and 5hmC can coexist at the same CpG site simultaneously on different strands of DNA [26], their distribution across the genome can overlap, reflecting a complex epigenetic landscape. The distribution of 5hmC varies widely among tissues, with the highest levels found in the central nervous system, particularly in neurons, followed by hematopoietic stem cells, pancreatic islets, and liver cells. This tissue-specific abundance suggests distinct regulatory roles for 5hmC across different biological contexts, which is further reflected by the variable expression patterns of TET enzymes [27]. Genome-wide mapping studies employing high-resolution sequencing techniques such as Tet-assisted bisulfite sequencing (TAB-seq) and oxidative bisulfite sequencing (oxBS-seq) have demonstrated that 5hmC is preferentially enriched within gene bodies, enhancers, and transcriptional start sites of actively transcribed genes. Presence of 5hmC is often associated with regions transitioning from repressive site to permissive chromatin states and active gene expression, contrasting with the typically repressive role of 5mC [62].

Although 5hmC is enriched in transcriptionally active regions, it also has distinct roles in heterochromatin, particularly within repetitive elements such as simple repeats, long and short interspersed nuclear elements (LINE and SINE) and LTRs. In general, its presence correlates positively with active histone marks such as H3K4me3 and H3K27ac that are associated with euchromatin. 5hmC can also associate with H3K27me3, that marks heterochromatin regions of the genome. Such associations are tissue-type dependent. Global levels of 5hmC and H3K27me3 have been shown to be co-regulated during cellular differentiation [23]. DNA immunoprecipitation sequencing (DIP-seq) analysis in wild-type and TDG deficient embryonic stem (ES) cells resulted in the identification of multiple regions were enriched in repetitive elements. While 5mC, 5fC and 5caC were present at major satellite repeats that are enriched at pericentric heterochromatin region, 5hmC was localized to LINEs, SINEs and LTRs [72]. Comparative analysis of genome wide cytosine modifications of repetitive elements in both mouse embryonic fibroblasts and ES cells also revealed distinct patterns of 5mC and 5hmC/5fC/5caC [61]. In ES cells, mouse-specific SINEs and LTRs were enriched for 5hmC, whereas in MEFs they accumulated more oxidized modifications (5fC and 5caC) when TDG was depleted, underscoring the influence of differentiation state on repeat modification patterns. TET1-dependent 5hmC accumulation occurs in the female germ cell chromocenters that represent aggregates of pericentric heterochromatin region. This accumulation of 5hmC at the chromocenters is lost in *Tet1* knockout mice [86]. Similarly, mouse ES cells lacking DNMT1 show an enrichment of 5hmC at the pericentromeric heterochromatin [25]. Conversion of 5mC to 5hmC has also been detected to the 3’ proximal region of some silenced promoters in the mouse brain by ME-Class2 based integrated epigenetic analysis [70]. Together, these findings highlight 5hmC as a dynamic epigenetic mark that not only modulates euchromatic gene activity but also regulates the structure and function of heterochromatic repeats in a cell type and context-dependent manner.

## Epigenetic interpreters of 5hmC

The regulatory roles of 5hmC are mediated through its recognition by specific 5hmC reader proteins, which translate this epigenetic mark into functional cellular outcomes. Various proteins such as MBD3, MeCP2, and UHRF2 act as readers of 5hmC. Methyl-CpG-binding domain (MBD) proteins, like MBD1, MBD2, and MBD4, exhibit reduced affinity for 5hmC compared to 5mC, studies suggest that some, like MBD3, may bind to 5hmC and play a role in regulating gene expression [13, 88]. MBD3 (methyl-CpG-binding domain protein 3) is a core component of the NuRD (nucleosome remodeling and deacetylase) complex. Unlike classical methyl-CpG-binding proteins that preferentially bind 5mC, MBD3 exhibits higher affinity for 5hmC-enriched regions, enabling it to interpret hydroxymethylation signals in the genome. Through its interaction with 5hmC, MBD3 recruits the NuRD complex to target loci, thereby modulating chromatin accessibility and fine-tuning transcriptional regulation. This function is particularly important in embryonic stem cells and differentiating tissues, where dynamic 5hmC patterns guide lineage-specific gene expression and repress pluripotency-associated genes[88]. Dysregulation of MBD3-mediated 5hmC recognition can perturb chromatin remodeling and transcriptional homeostasis, highlighting its critical role in linking epigenetic modifications to cellular identity and developmental programs. MeCP2 (Methyl-CpG-binding Protein 2) is a well-characterized member of the MBD protein family that primarily binds to 5mC, but it can also recognize 5hmC in specific genomic contexts. This dual recognition allows MeCP2 to act as a versatile regulator of chromatin structure and gene expression [5, 57]. In neurons, where 5hmC levels are particularly high, MeCP2 binding to hydroxymethylated regions contributes to transcriptional modulation of genes involved in synaptic function, neuronal development, and activity-dependent plasticity. Importantly, MeCP2 recruits histone deacetylase (HDAC)–containing co-repressor complexes to 5hmC-enriched loci, forming the MeCP2–HDAC repressor complex, which can fine-tune chromatin accessibility and transcriptional repression in a context-dependent manner [89]. Dysregulation of MeCP2, including altered recognition of 5hmC or misfunction of the MeCP2–HDAC complex, is implicated in neurodevelopmental disorders such as Rett syndrome, highlighting the critical role of MeCP2 as a reader of hydroxymethylation in maintaining proper neuronal gene expression and genomic stability [20].

UHRF2 (ubiquitin-like with PHD and RING finger domains 2) functions as a key reader of 5hmC and plays an important role in epigenetic regulation. Unlike UHRF1, which preferentially recognizes 5mC and maintains DNA methylation during replication, UHRF2 contains an SRA (SET- and RING-associated) domain with a higher affinity for 5hmC [90]. UHRF1 can bind to TET1s, a smaller isoform of TET1 lacking the N-terminal Zinc-finger domain and recruit it to replicating heterochromatin. TET1s bound to UHRF1 oxidizes 5mC to 5hmC and results in chromatin decondensation and activation of LINE1 elements [4]. On the other hand, UHRF2 specifically binds hydroxymethylated CpG sites and recruits additional chromatin-modifying complexes to regulate gene expression. This selective recognition of 5hmC links UHRF2 to processes such as transcriptional activation, cell cycle progression, and differentiation. Importantly, UHRF2-mediated stabilization of 5hmC contributes to epigenetic memory and genome integrity [8, 81]. Dysregulation of UHRF2 has been implicated in cancer, where alterations in its ability to recognize and interpret 5hmC may contribute to disrupted gene expression and tumor progression [52]. Thus, UHRF2 acts as a crucial interpreter of hydroxymethylation, translating the 5hmC mark into functional outcomes in both normal physiology and disease contexts. In summary, MBD3, MeCP2, and UHRF2 serve as key readers of 5hmC, translating this dynamic epigenetic mark into functional outcomes across different cellular contexts. Together, these readers highlight the diverse and context-specific mechanisms by which 5hmC shapes transcriptional programs, cellular identity, and genome maintenance in both normal physiology and disease.

## Role of 5hmC in normal cellular processes

5hmC plays a multifaceted role in regulating key cellular processes. 5hmC is not just a passive intermediate of DNA demethylation, it actively regulates transcription, epigenetic reprogramming, development, neuronal gene expression, genome stability, and cell cycle control in normal physiology. In comparison, 5mC maintains stable gene silencing and genomic integrity in normal biological processes, while 5hmC is linked to active gene regulation, especially during development and cell differentiation. Unlike 5mC, which promotes long-term repression, 5hmC marks dynamic regions and supports transcriptional flexibility. Throughout development and cellular differentiation, dynamic remodeling of the 5hmC landscape plays a critical role in guiding lineage specification by activating lineage-specific gene programs while concurrently repressing genes associated with pluripotency, thereby ensuring proper cell fate commitment and tissue-specific function.

In embryonic stem cells (ESCs), elevated levels of TET1 and 5hmC maintain a poised chromatin state that permits rapid transcriptional activation upon differentiation cues [15, 71]. Similarly, in hematopoietic stem cells, TET2 and 5hmC are critical for preserving stemness and guiding lineage commitment [14, 40]. In the nervous system, 5hmC is especially enriched and contributes significantly to neurodevelopment, synaptic plasticity, and memory formation [41, 68, 75]. Its localization to neuron-specific gene bodies and active enhancers supports its role in long-term gene regulation. Perturbations in 5hmC distribution have been linked to neurodevelopmental and neurodegenerative disorders, highlighting its importance in brain function [9, 39]. Additionally, 5hmC and TET proteins are involved in the regulation of the cell cycle and the DNA damage response. Fluctuations in 5hmC levels during the cell cycle correlate with the expression of key regulatory genes, while TET enzymes have been shown to interact with components of DNA repair pathways. These interactions suggest a role for 5hmC in maintaining genomic stability and proper cell cycle progression [10, 35]. Studies have shown that TET deficiency can lead to cell cycle defects, such as centrosome abnormalities and altered cyclin B1 levels [10]. Moreover, 5hmC has been shown to accumulate at sites of DNA damage and repair, suggesting a role for TET enzymes and 5hmC as epigenetic markers in the DDR pathway. Specifically, TET3-mediated DNA oxidation in response to ATR-dependent DDR has been implicated in regulating DNA repair mechanisms [33]. Loss of TET enzymes and the resulting depletion of 5hmC can impair damage-induced 5hmC accumulation, leading to chromosome segregation defects and genome instability [17]. 5hmC influences replication fork stability, particularly via recruiting apurinic/apyrimidinic (AP) endonuclease, APE1 for degradation at stalled forks, impacting genomic integrity [36, 38]. This underscores the importance of TET proteins and 5hmC in safeguarding genome integrity and suggests that their dysregulation can contribute to tumorigenesis and other diseases.

## 5hmC in carcinogenesis and cancer progression

**Fig. 1:**
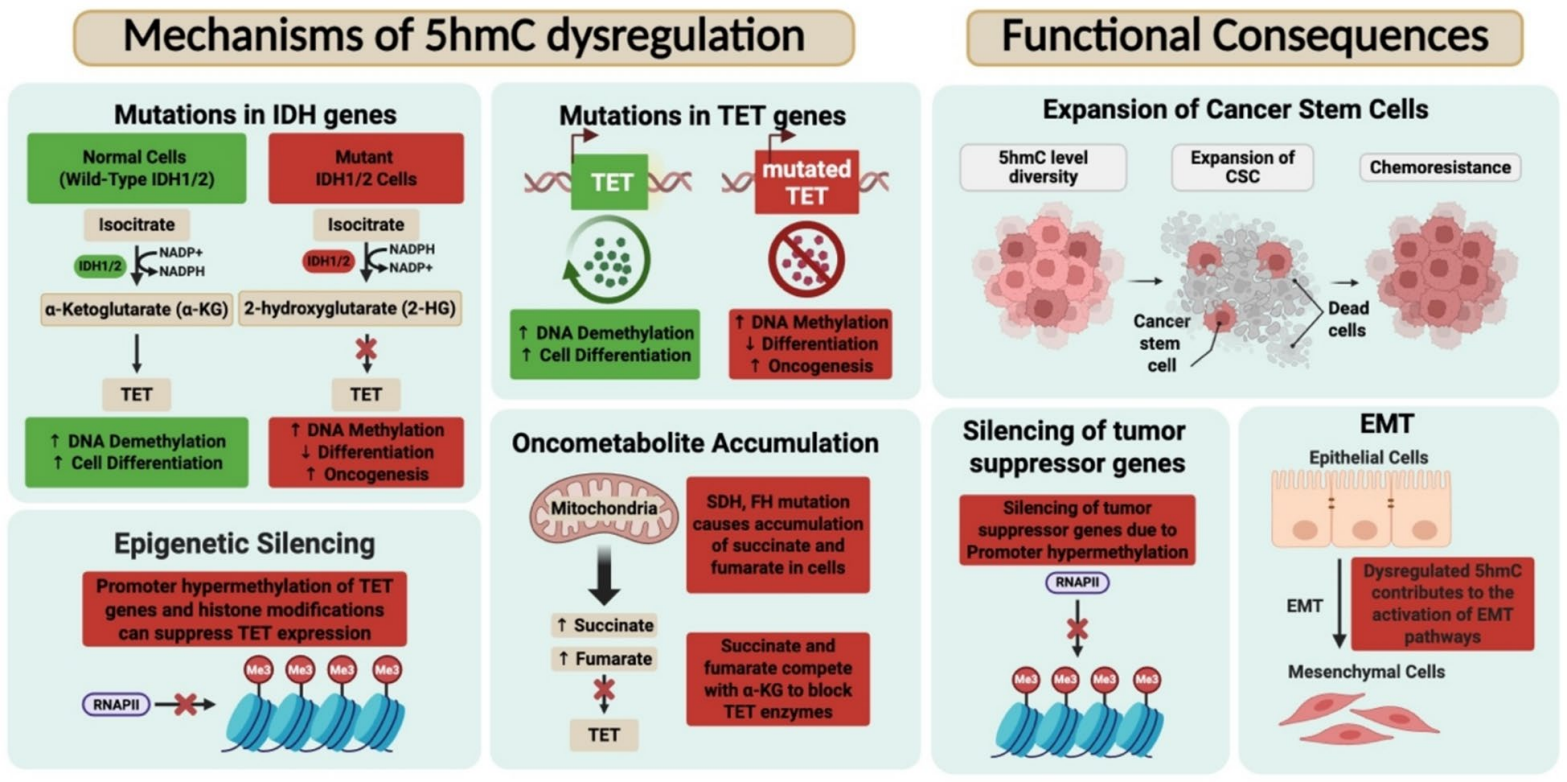
Mechanisms and functional consequences of 5hmC dysregulation in cancer. This figure illustrates the molecular mechanisms leading to 5hmC dysregulation and its downstream functional consequences across malignancies. At the core of 5hmC regulation are the TET family of enzymes (TET1, TET2, TET3), which catalyze the conversion of 5mC to 5hmC. Mutations or epigenetic silencing of TET genes, accumulation of oncometabolites such as 2-HG, fumarate, and succinate from mutations in IDH1/2, FH, and SDH genes, respectively, as well as oxidative or metabolic stress, can impair TET activity, leading to global or gene-specific loss of 5hmC. These oncometabolites competitively inhibit α-ketoglutarate-dependent dioxygenases, including TET enzymes, thereby blocking DNA demethylation and promoting oncogenic epigenetic reprogramming. Functionally, 5hmC loss is associated with altered enhancer activity, disrupted chromatin accessibility, impaired transcriptional regulation, and silencing of tumor suppressor genes. These epigenetic changes contribute to malignant progression by promoting expansion of cancer stem cell populations, inducing epithelial-to-mesenchymal transition (EMT), and facilitating therapeutic resistance.

It is now well established that 5hmC plays a distinct role in the complex landscape of carcinogenesis and cancer progression, transcending its initial perception as merely an intermediate in DNA demethylation. DNA methylation involving 5mC and 5hmC plays distinct yet interconnected roles in the context of carcinogenesis and cancer progression. Aberrant patterns of 5mC, including global hypomethylation and promoter-specific hypermethylation of tumor suppressor genes, are characteristic of cancer. These alterations contribute to the activation of oncogenes and the silencing of tumor suppressor genes. On the other hand, 5hmC is often enriched in actively transcribed regions and plays a role in gene expression and maintaining genomic stability. Figure 1 highlights important mechanisms and functional consequences of 5hmC dysregulation in cancer. One of the most consistent and striking observations in cancer epigenetics is the global reduction of 5hmC across a broad range of tumor types, including glioblastoma, melanoma, colorectal, breast, and hematological malignancies [22, 46, 65]. This widespread loss of 5hmC is closely associated with poor cellular differentiation, heightened proliferative capacity, and worse clinical outcomes.

Multiple mechanisms contribute to 5hmC dysregulation in cancer, with alterations in TET enzyme function playing a central role. Loss-of-function mutations in TET2, frequently observed in myeloid malignancies, result in diminished 5hmC levels and impaired hematopoietic differentiation [40]. Similarly, mutations in IDH1 and IDH2 lead to the accumulation of the oncometabolite 2-hydroxyglutarate (2-HG), which acts as a competitive inhibitor of TET enzymes, further reducing 5hmC abundance [85, 87]. In addition to genetic alterations, epigenetic silencing, such as promoter hypermethylation and repressive histone modifications, can suppress TET gene expression [19, 44]. Furthermore, metabolic changes within the tumor microenvironment, including fluctuations in the levels of essential TET cofactors like α-ketoglutarate (α-KG), succinate, and fumarate, can modulate TET activity and thereby reshape the epigenetic landscape [32, 80]. The functional consequences of impaired TET activity in tumor biology are profound, as the resulting failure to initiate active DNA demethylation leads to sustained promoter hypermethylation and transcriptional silencing of tumor suppressor genes. In this context, the observed reduction in 5hmC levels primarily reflects a loss of TET-mediated oxidation activity, with 5hmC serving as an intermediate in the demethylation process rather than a direct epigenetic effector. Enhanced cellular stemness is another hallmark, as low 5hmC levels support the expansion and maintenance of cancer stem-like cells, which are often more resistant to conventional therapies [6, 21, 79]. Additionally, epithelial-mesenchymal transition (EMT) pathways become aberrantly activated under 5hmC-deficient conditions, promoting increased tumor cell invasion and metastasis [19].

5hmC dysregulation has been implicated across multiple cancer hallmarks. Global loss and locus-specific redistribution of 5hmC can sustain proliferative signaling by activating oncogenes and silencing tumor suppressors, while also contributing to apoptosis resistance through preferential regulation of pro-survival pathways. Altered 5hmC landscapes may further support replicative immortality by stabilizing stemness-related gene expression and promote invasion and metastasis via epigenetic activation of epithelial–mesenchymal transition (EMT) drivers. In addition, 5hmC changes influence genome stability and DNA damage response, enabling tumor cells to tolerate genotoxic stress. Collectively, these aberrant 5hmC patterns provide a versatile epigenetic framework that reinforces diverse cancer hallmarks. Table 1 summarizes the verified and speculative roles of 5hmC in cancer hallmarks. Collectively, these findings highlight pivotal role of TET proteins and 5hmC as a critical epigenetic regulator whose disruption not only marks cancer development but also actively drives tumor progression through its multifaceted influence on cancer hallmarks.

**Table 1:** Role of 5hmC in regulating cancer hallmarks

Cancer hallmark	Proven role of 5hmC	Speculative/unverified
Proliferation	Global loss of 5hmC in non-proliferating tumor cells [64]	Effect of locus specific 5hmC loss on growth is not yet demonstrated
Growth suppressor evasion	Ectopic expression of TET1 inhibited proliferation in ovarian cancer via RASSF5 [43]	The broader role of 5hmC in enabling tumor suppressor pathways is hypothesized
Apoptosis resistance	In hepatocellular carcinoma, low 5hmC reduces apoptosis and promotes proliferation [21]	5hmC redistribution may foster apoptosis resistance by activating survival pathways
Replicative immortality	Global depletion of 5hmC correlates with high proliferation in tumor cells [64]	Direct connection to telomerase activation or bypass of senescence hasn’t been shown
Angiogenesis	None	Epigenetic regulation of angiogenic genes mediated by 5hmC changes is not yet proven
Invasion and metastasis	Reintroduction of TET2 (restoring 5hmC) in melanoma cells decreased metastatic potential [46]	Direct locus-specific 5hmC changes at invasion-related genes remain to be mapped
Phenotypic plasticity	Low 5hmC is correlated to enrichment of cancer stem like cells in HCC [21]	Direct link between 5hmC and stemness transcriptional programs remains to be established
Epigenetic reprogramming	Loss of 5hmC across many tumor types affects epigenetic reprogramming [46]	Locus specific effect of 5hmC of epigenetic reprogramming is unclear
Microbiome influence	None	Microbial metabolites affecting TET enzymes remains to be untested
Senescence	None	Since senescence involves epigenetic changes, involvement of 5hmC is plausible but unstudied
Genome instability	TET2 mutations (loss of 5hmC) in myeloid malignancies and linked to genomic instability [54]	Direct link between 5hmC loss and mutation accumulation is not yet proven
Tumor-promoting inflammation	None [78]	Loss of 5hmc due to hypoxia and microenvironmental may influence inflammation
Avoiding immune destruction	None	Epigenetic regulation of immune checkpoints by 5hmC remains an open hypothesis

## 5hmC as a biomarker in cancer diagnosis and prognosis

5hmC has emerged as a promising diagnostic biomarker because of its unique biochemical stability, cancer-specific alterations, and feasibility of detection in both tissue specimens and non-invasive liquid biopsies. While both 5mC and 5hmC are crucial epigenetic marks with roles in cancer, their utility as biomarkers in cancer diagnosis and prognosis presents distinct characteristics. 5mC, particularly hypermethylation in gene promoter regions, serves as a well-established biomarker for early cancer detection and stratification, indicating gene silencing events common in various malignancies. However, the global reduction of 5hmC, driven by impaired TET enzyme activity, has emerged as another significant and often more specific biomarker in numerous cancer types. In cancer biology, global depletion of 5hmC is a hallmark epigenetic event that reflects widespread DNA methylation reprogramming during tumorigenesis. Such loss has been consistently reported in multiple solid tumors, including melanoma, glioblastoma, breast, prostate, and colorectal cancers, where it distinguishes malignant from normal tissues and highlights the disruption of TET-mediated hydroxymethylation pathways. Table 2 summarizes the prognostic and diagnostic relevance of 5hmC levels in various cancers. Beyond global depletion, 5hmC changes are tissue- and cancer-type specific, creating distinct epigenetic signatures that enable accurate classification of tumor origins and histological subtypes [74]. This specificity gives 5hmC a unique advantage over other epigenetic marks, which often lack such discriminatory power. At the histological level, immunohistochemical staining for 5hmC has proven particularly useful, as it allows visualization of its progressive loss across cancer development. For example, in melanoma, a gradual decline in 5hmC can be observed from benign nevi to invasive and metastatic tumors [66]. This stepwise reduction provides a powerful tool to differentiate between early-stage and advanced disease. Similarly, in pleural mesothelioma, immunostaining for 5hmC has demonstrated high diagnostic accuracy in separating malignant proliferations from benign mesothelial hyperplasia, conditions that are otherwise notoriously difficult to distinguish morphologically.

**Table 2:** Diagnostic/prognostic significance of 5hmC in human cancers

Cancer type	Diagnostic/prognostic role	Therapeutic implication	References
Hepatocellular Carcinoma	Loss of 5hmC linked to poor prognosis and recurrence	Low 5hmC → PCAF suppression → AKT hyperactivation → Chemoresistance	[21]
Prostate Cancer	Elevated cfDNA 5hmC levels at AR, FOXA1, and GRHL2 genes are indicative of androgen deprivation therapy resistance.	Stratifies patients for early intervention	[45]
ESCC (Esophageal)	cfDNA 5hmC linked to metastasis, recurrence	Non-invasive monitoring tool	[42, 51] [51]
CLL	5hmC profiling distinguishes between indolent and aggressive forms	Prognostic utility in treatment planning	[82]
Breast cancer	TET2/5hmC loss correlates with BRCA1/2-deficient chemoresistance	Vitamin C restores 5hmC and PARP inhibitor sensitivity	[36, 37]
Cholangiocarcinoma	Reduced 5hmC linked to IDH mutations, used in diagnostic panels	IDH inhibitors like ivosidenib show benefit via 5hmC restoration	[1]
Glioma	5hmC generally preserved in IDH-wildtype, reduced in IDH-mutant tumors	Targeting IDH mutation with inhibitors can restore 5hmC and enhance response	[18, 59]
AML	Distinct 5hmC signatures serve as predictive biomarkers for therapy response	5hmC patterns guide azacitidine/chemo response prediction	[47]

In liquid biopsy applications, circulating cell-free DNA (cfDNA) profiling of 5hmC offers a minimally invasive alternative for cancer detection and monitoring. Technical advances now permit sensitive mapping of 5hmC signatures in cfDNA, uncovering tumor-specific epigenetic patterns that reflect the tissue of origin. For instance, distinct 5hmC cfDNA profiles have been identified in breast cancer and hepatocellular carcinoma, enabling both diagnosis and disease classification without tissue sampling [3, 7]. In esophageal squamous cell carcinoma (ESCC), cfDNA-based 5hmC signatures extend this diagnostic value by also correlating with metastatic spread and recurrence risk. This makes them clinically relevant for longitudinal monitoring, predicting relapse, and guiding therapeutic decision-making during follow-up [42, 51]. Although global 5hmC levels are frequently reduced in cancer tissues, this does not preclude its utility as a biomarker in cfDNA. Evidence suggests that 5hmC is not uniformly lost but rather redistributed across the genome, resulting in cancer-specific 5hmC patterns. These distinct patterns can be captured in circulating cfDNA, enabling non-invasive detection of tumors and monitoring of minimal residual disease (MRD). Therefore, the observed global loss of 5hmC and the effectiveness of cfDNA 5hmC-based liquid biopsy are not contradictory; rather, they reflect the selective redistribution of 5hmC marks in cancer cells that provides a unique molecular fingerprint for diagnostic and prognostic purposes. Cholangiocarcinoma is often associated with reduced levels of 5hmC, particularly in cases harboring isocitrate dehydrogenase (IDH) mutations. This epigenetic alteration has been incorporated into diagnostic panels to improve detection and stratification of patients. Targeted therapies such as IDH inhibitors, including ivosidenib, have shown clinical benefit by restoring 5hmC levels, thereby counteracting the epigenetic dysregulation and offering a precision-medicine approach to treatment [1]. Collectively, these studies establish 5hmC as a clinically actionable diagnostic tool with broad utility across both tissue- and cfDNA-based assays.

In addition to its diagnostic applications, 5hmC serves as a powerful prognostic biomarker, providing insights into disease aggressiveness, survival outcomes, and therapy responsiveness. A consistent theme across cancer studies is that reduced global levels of 5hmC correlate with poor clinical outcomes, including aggressive histopathological features, poor differentiation, higher tumor grade, greater metastatic potential, and shortened patient survival. This association has been documented in multiple solid tumors, such as melanoma and glioma, where global 5hmC loss reflects an underlying failure of epigenetic regulation critical for maintaining tumor cell identity and genomic stability [46, 48, 59]. Conversely, preservation or partial restoration of 5hmC appears to be a favorable prognostic indicator. In gliomas and myeloid malignancies, patients with higher residual levels of 5hmC demonstrate significantly better survival outcomes [34]. Mechanistically, this may reflect the role of 5hmC in sustaining transcriptional programs that limit tumor proliferation and promote cellular differentiation. Restoration of 5hmC, either naturally or through therapeutic strategies such as vitamin C supplementation that enhances TET enzyme activity, has been shown to improve responsiveness to treatment in experimental models [37].

In chronic lymphocytic leukemia (CLL), 5hmC profiling has shown potential to distinguish between indolent and aggressive disease subtypes, reflecting underlying epigenetic differences that drive clinical heterogeneity. Reduced or altered 5hmC patterns are often associated with more aggressive progression, whereas preserved 5hmC correlates with slower disease course. This prognostic utility is valuable in treatment planning, as it helps identify patients who may benefit from early therapeutic intervention versus those suitable for a watch-and-wait strategy, thereby supporting more personalized management of CLL [82]. In hematological malignancies, the prognostic implications of 5hmC are further shaped by the mutational status of TET2. TET2 mutations result in profound reductions of 5hmC, which are linked to adverse outcomes in disorders such as acute myeloid leukemia (AML) and myelodysplastic syndromes. However, the prognostic utility of 5hmC levels in specific subtypes, such as cytogenetically normal karyotype AML, remains an active area of investigation [2, 60].

Importantly, these studies underscore that 5hmC does not act as a uniform biomarker across all cancers but instead provides nuanced, context-dependent prognostic information. Ultimately, assessment of 5hmC offers a complementary layer of prognostic value alongside traditional clinical and molecular factors. By stratifying patients into distinct risk categories based on their epigenetic landscape, 5hmC profiling could guide more personalized treatment strategies. For example, patients with persistently low 5hmC may require intensified therapy and closer monitoring, whereas those with preserved 5hmC might benefit from standard regimens with reduced risk of relapse. Thus, the prognostic relevance of 5hmC lies not only in its correlation with survival outcomes but also in its capacity to refine therapeutic decision-making and improve individualized cancer care. Ultimately, 5hmC serves as both a diagnostic and prognostic biomarker, with its tissue- and cancer-specific distribution enabling accurate tumor classification and its dynamic levels correlating with patient outcomes. Detectable in tissue and liquid biopsies, 5hmC offers a non-invasive means for early detection, monitoring, and risk stratification. By linking diagnostic precision with prognostic value, 5hmC holds strong translational potential to guide personalized cancer management and improve clinical outcomes.

## Mechanisms linking 5hmC with chemotherapy response

The interplay between 5hmC and chemotherapy response has become a focus of considerable research interest due to its potential to influence therapeutic efficacy across various cancer types. One of the well-documented observations is the loss of 5hmC in hepatocellular carcinoma (HCC), where reduced 5hmC levels, regulated by TET2, promote chemoresistance. This resistance is mediated through suppression of the histone acetyltransferase P300/CBP-associated factor (PCAF), resulting in hyperactivation of the AKT signaling pathway. This 5hmC/PCAF/AKT axis illustrates a critical epigenetic mechanism driving drug resistance in HCC [21]. Loss of TET2 has been shown to confer resistance to Poly(ADP-ribose) polymerase (PARP) inhibitors (PARPi) in breast cancer cells, primarily by enhancing replication fork protection, thereby allowing cells to better tolerate DNA damage [36]. Conversely, pharmacological activation of TET enzymes using Vitamin C leading to global increase in 5hmC levels can exacerbate PARP trapping at sites of DNA damage, effectively restoring sensitivity to PARPi in chemoresistant cancer cells, particularly those harboring BRCA1 or BRCA2 deficiencies. This highlights a potential therapeutic strategy to overcome acquired resistance to PARP inhibition by modulating 5hmC levels [37].

In acute myeloid leukemia (AML), specific 5hmC signatures have been identified as predictive biomarkers for therapeutic response. Patients with favorable 5hmC patterns showed improved outcomes when treated with azacitidine in combination with standard chemotherapy, highlighting the prognostic value of 5hmC profiling in hematological malignancies [47]. In prostate cancer, 5hmC patterns in cfDNA have been associated with resistance to androgen deprivation therapy (ADT). Patients with hypermethylation of genes involved in AR, FOXA1, and GRHL2 pathways prior to therapy were more likely to develop resistance, suggesting a predictive role for 5hmC in guiding early therapeutic decisions [45]. Across multiple cancer types, reduced global 5hmC levels are consistently associated with aggressive disease and unfavorable clinical outcomes (Table 2). While loss of 5hmC can contribute to drug resistance, these mechanistic effects remain less clearly defined compared to its established correlation with therapeutic outcomes. This predictive value may reflect the broader functional status of TET-mediated demethylation, with reduced 5hmC serving as a surrogate for epigenetic dysregulation affecting drug response pathways. Thus, 5hmC has a strong potential as a clinically useful predictive biomarker for patient stratification. Its mechanistic role in resistance, though secondary, offers an additional biological context. Future directions include the development of therapeutic strategies aimed at restoring 5hmC levels, either through activation of TET enzymes or inhibition of metabolic pathways contributing to 5hmC loss. Integrating 5hmC profiling into clinical practice may pave the way for more personalized and effective cancer treatment approaches. The interplay between 5hmC and chemotherapy response has garnered significant attention in recent years. Emerging studies have elucidated various mechanisms through which alterations in 5hmC levels influence chemotherapeutic efficacy across different cancer types.

## Therapeutic targeting of 5hmC modifiers

Targeting TET enzymes and 5hmC hold significant therapeutic potential in cancer, with several strategies under investigation. Small molecules that enhance TET activity or promote 5hmC levels, such as vitamin C, have shown promise in preclinical studies. Promoting TET activity and restoring 5hmC levels in cancer cells, potentially sensitizes them to chemotherapy [45, 63, 69, 73]. PARP inhibitors are effective in cancers with homologous recombination (HR) defects, particularly BRCA1/2-mutated tumors. PARPis such as olaparib, talazoparib, rucaparib, and niraparib have received FDA approval for treatment of multiple different cancers such as ovarian, breast, pancreatic, and prostate [24]. By exploiting synthetic lethality, PARP inhibitors selectively kill HR-deficient cells. Their cytotoxicity arises not only from blocking PARP enzymatic activity in single-strand break repair but also from PARP trapping, where trapped PARP1 stalls replication forks, causing genomic instability and cell death [50, 58, 67]. Recent studies have identified vitamin C (ascorbate), a cofactor for TET dioxygenases, as a promising epigenetic modulator capable of restoring 5hmC levels in PARPi-resistant cancer cells. This restoration of 5hmC has been shown to enhance PARP1 trapping, thereby re-sensitizing chemoresistant BRCA mutated cancer cells to PARP inhibition. These findings highlight the therapeutic potential of combining epigenetic reprogramming strategies with PARPi to overcome acquired resistance in HR-deficient cancers [37]. Targeting 2'-Deoxynucleoside 5'-Phosphate N-Hydrolase 1 (DNPH1), an enzyme that clears hmdU generated during 5hmC processing, heightens PARPi sensitivity in BRCA-deficient cells by promoting DNA damage. This strategy also helps overcome PARPi resistance, highlighting its therapeutic potential [16]. IDH inhibitors, like ivosidenib and enasidenib, offer a targeted approach to reverse the inhibitory effect of 2-HG, an oncometabolite that accumulates in IDH-mutant cancers and inhibits TET enzymes, thereby restoring 5hmC levels and potentially improving outcomes, particularly in IDH-mutant AML [12, 53, 85]. Combination epigenetic therapies, such as using hypomethylating agents in combination with histone deacetylase inhibitors, are being investigated for their potential to reshape the cancer epigenome and improve treatment responses. In addition, therapies that activate TET enzymes or are combined with immune checkpoint inhibitors may further enhance clinical outcomes [56, 76].

Emerging CRISPR and epigenome editing technologies, particularly the use of catalytically inactive Cas9 (dCas9) fused to epigenetic modifiers like TET enzymes (e.g., dCas9-TET1), provide a highly precise strategy to manipulate 5hmC levels at specific genomic loci. By guiding the dCas9-TET fusion protein to defined DNA regions via sequence-specific single guide RNAs (sgRNAs), this approach enables locus-specific oxidation of 5mC to 5hmC without inducing double-strand breaks. Such targeted epigenetic editing holds great promise for reactivating silenced tumor suppressor genes or modulating oncogene expression in a controlled manner. Studies have demonstrated that this system can reprogram aberrant DNA methylation landscapes in cancer cells, restore transcriptional activity of key regulatory genes, and potentially overcome drug resistance. This platform thus represents a powerful therapeutic tool for precise reactivation of critical gene networks involved in tumor suppression, cell differentiation, or immune response [49].

## Challenges in 5hmC research

Despite significant advances in understanding the role of 5hmC in cancer, several challenges hinder progress in this field. Technical limitations remain, as high-resolution 5hmC mapping is costly and requires specialized expertise. The complex interplay between 5mC, 5hmC, and histone modifications further complicates the interpretation of epigenetic data, making it difficult to isolate the specific contributions of 5hmC. Additionally, intratumoral heterogeneity in 5hmC distribution poses major obstacles for consistent biomarker identification and validation, limiting the reliability of clinical applications. Finally, global modulation of epigenetic enzymes introduces the risk of off-target effects and unintended cellular reprogramming, raising significant concerns about the safety and precision of approaches that alter 5hmC patterns.

## Emerging insights and therapeutic prospects

Major open questions about 5hmC in cancer and cancer therapy cluster around mechanism, timing, biomarker utility, therapeutic manipulability, functional heterogeneity, and even its potential role in broader cancer hallmarks. It is well established that many solid tumors and aggressive malignancies show a broad loss or redistribution of 5hmC compared with matched normal tissues, a reproducible epigenomic hallmark that has diagnostic and prognostic implications. Clinically promising work shows that 5hmC patterns in circulating cfDNA can discriminate cancer from non-cancer and correlate with outcome in several settings, yet we still need prospective, multicenter evidence that 5hmC-based assays improve screening, early detection, or management beyond existing modalities and to define standardized, robust workflows for clinical deployment. Translationally, several epigenetic therapies (for example DNMT inhibitors) and co-factors such as vitamin C can modulate TET activity and alter 5hmC landscapes in cell and animal models, suggesting that therapeutic re-establishment of 5hmC is possible, but whether restoring 5hmC per se produces durable anti-tumor effects, or instead generates context-dependent reprogramming with mixed outcomes, remains unsettled. It is plausible that locus-specific restoration of 5hmC at enhancers or gene bodies rather than global increases will be required to produce beneficial transcriptional reprogramming without unintended activation of oncogenic programs, but targeted delivery or locus-specific epigenetic editing tools to achieve that in patients are still at an early conceptual stage and have not been validated in clinical models. Another open question is how 5hmC is involved in DNA repair, genome stability, and immunogenicity in tumors. Emerging preclinical work hints that altered cytosine oxidation products modify repair pathway choice and nucleosome dynamics, and may change exposure of immunogenic DNA motifs, if so, combining TET-modulating agents with DNA-damage therapies or immunotherapy could be synergistic, but this hypothesis requires rigorous mechanistic and safety studies. Additional pressing questions concern heterogeneity and timing: how early in tumor evolution 5hmC changes occur, whether they are reversible in long-lived cancer stem/progenitor compartments, and how intratumoral and interpatient heterogeneity of 5hmC landscapes predicts therapeutic response or resistance. Finally, from a methods perspective, standardized, high-resolution assays that distinguish true 5hmC from other oxidized cytosine species in low-input clinical samples (e.g., small biopsies, cfDNA) are still improving. Better assays will be essential to translate mechanistic insight into validated biomarkers or to guide trials of agents that aim to modulate TET/5hmC biology. Beyond these established questions, a particularly intriguing but underexplored area is whether 5hmC contributes to cancer hallmarks that currently lack direct experimental links. For example, alterations in 5hmC at enhancer regions could promote metabolic reprogramming by shifting transcription of metabolic enzymes, aligning with the hallmark of deregulated cellular energetics. Similarly, 5hmC-mediated chromatin changes may influence immune evasion, not only by altering antigen presentation pathways but also by reshaping tumor–immune crosstalk through cytokine regulation. Another possible role is in enabling replicative immortality, as dynamic cytosine oxidation states at telomere-associated loci could theoretically influence telomerase regulation and chromatin architecture. Finally, changes in 5hmC distribution might contribute to promoting tumor-promoting inflammation or sustaining proliferative signaling indirectly, by fine-tuning enhancer accessibility of key transcription factors. Together, these open questions underscore that the role of 5hmC in cancer is still far from fully resolved, and future work integrating mechanistic, biomarker, and therapeutic studies will be essential to determine whether targeting 5hmC can move from a conceptual promise to a clinically actionable reality.

## Data Availability

No datasets were generated or analyzed during the current study.

## Abbreviations

5mC: 5-Methylcytosine
5hmC: 5-Hydroxymethylcytosine
5caC: 5-Carboxylcytosine
5fC: 5-Formylcytosine
TET: Ten-eleven translocation
IDH: Isocitrate dehydrogenase
2-HG: 2-Hydroxyglutarate
PARPi: Poly(ADP-ribose) polymerase (PARP) inhibitors
α-KG: α-Ketoglutarate
TDG: Thymine DNA glycosylase
DNPH1: Deoxynucleoside 5'-Phosphate N-Hydrolase 1
AML: Acute myeloid leukemia
cfDNA: Cell free DNA
TAB-seq: Tet-assisted bisulfite Sequencing
OxBS-seq: Oxidative bisulfite sequencing
